# Case Report: Pediatric immune-mediated necrotizing myopathies mimicking inherited muscle disorders: clinical, paraclinical, and genetic insights from two cases

**DOI:** 10.3389/fped.2026.1906576

**Published:** 2026-08-28

**Authors:** Gianmarco Severa, Maria Camila Cortes-Rojas, Marta Gomez-Garcia de la Banda, Thibaut Belmondo, Audrey Benezit, France Leturcq, Camille Verebi, Baptiste Periou, Eduard Gallardo, Denise Cassandrini, Thierry Gendre, Sophie Hue, Cyril Gitiaux, Nadia Toubal, Sonia Nouioua, Nadira Boucher, Brigitte Bader-Meunier, François Jérôme Authier, Robert-Yves Carlier, Susana Quijano-Roy, Edoardo Malfatti

**Affiliations:** 1 University Paris Est Créteil, Inserm, U955, IMRB, Créteil, France; 2 Reference Center for Neuromuscular Disorders, APHP Henri Mondor University Hospital, Créteil, France; 3 Child Neurology Department, Hospital Militar Central, Bogotá, Colombia; 4 Neuromuscular Center, Child Neurology and ICU Department, Raymond Poincare Hospital, AP-HP, GHU Université Paris-Saclay, Garches, France; 5 Département d’Immuno-Hématologie, GHU AP-HP, Hôpitaux Universitaires Henri-Mondor, APHP, Créteil, France; 6 Service de Médecine Génomique des Maladies de Système et d'Organe, Fédération de Génétique et de Médecine Génomique, APHP, Centre—Université Paris Cité, Hôpital Cochin, Paris, France; 7 Department of Neurology, Neuromuscular Diseases Unit, Hospital de la Santa Creu i Sant Pau, Universitat Autònoma de Barcelona, Barcelona, Spain; 8 Biomedical Research Institute Sant Pau (IIB Sant Pau), Barcelona, Spain; 9 Biomedical Network Research Centre on Rare Diseases (CIBERER), Madrid, Spain; 10 Immunology Unit, University Hospital, Azienda Ospedaliera Universitaria Integrata, Verona, Italy; 11 Department of Neurosciences, Biomedicine and Movement Sciences, University of Verona, Verona, Italy; 12 Neurology Department, APHP Henri Mondor University Hospital, Créteil, France; 13 Reference Center for Neuromuscular Disorders, Filnemus, EuroNMD, Assistance Publique-Hôpitaux de Paris (APHP) Necker Enfants Malades Hospital, Paris, France; 14 Department of Neurology, CHU IBN ROCHD, Faculté de Médecine, Université BADJI MOKHTAR, Annaba, Algeria; 15 Department of Neurology, Cherchell EHS, Tipaza, Algeria; 16 Faculty of Medicine, Department of Pediatrics, ANNABA University Hospital, Badji Mokhtar University, Annaba, Algeria; 17 Department for Immunology, Hematology and Pediatric Rheumatology, Necker Hospital, APHP, Institut IMAGINE, Paris, France; 18 APHP, Université UVSQ-Paris Saclay, Hôpital Raymond Poincaré, Service de Radiologie, Garches, France

**Keywords:** case report, immune-mediated necrotizing myopathy, muscle biopsy, seronegative, SRP antibodies

## Abstract

**Objectives:**

Immune-mediated necrotizing myopathies (IMNMs) are a group of rare acquired myopathies. We describe two pediatric cases presenting with early-onset proximal and axial muscle weakness, rhabdomyolysis, and progressive evolution mimicking limb-girdle muscular dystrophy.

**Methods:**

This retrospective case series is based on a review of the medical records of two pediatric IMNM patients with symptom onset at 2 (P1) and 16 (P2) years of age and diagnoses at 5 and 17 years, respectively. Both patients underwent clinical assessment, muscle biopsy, whole-body muscle MRI, a testing for myositis-specific and myositis-associated autoantibodies, whole-exome sequencing, and *in silico* analysis following informed parental consent. These data were collected and analyzed retrospectively. The manuscript was prepared in accordance with the CARE reporting guidelines.

**Results:**

Both patients presented with an early onset of severe muscle weakness, compatible with the clinical suspicion of limb-girdle muscular dystrophy. P1’s serological screening was positive for anti-signal recognition particle antibodies, and muscle biopsy revealed a myopathological pattern compatible with IMNM. Treatment with corticosteroids, intravenous immunoglobulin (IVIG), and rituximab resulted in partial amelioration of muscle strength. P2’s screening for myositis autoantibodies was negative. Muscle biopsy showed a dystrophic pattern with the absence of dysferlin. Whole-exome sequencing did not identify any compatible variants explaining the muscle phenotype in both patients. P2 was treated with corticosteroids, IVIG, and rituximab, resulting in normalization of muscle weakness and creatine kinase (CK) levels.

**Conclusion:**

IMNM should be considered in pediatric patients with clinical and paraclinical suspicion of inherited muscular dystrophy. Serological testing is crucial for identifying seropositive cases. In the presence of seronegative or more complex cases, a global paraclinical and genetic analysis is essential to avoid misdiagnosis and initiate long-term immunosuppressive therapy, including corticosteroids, IVIG, and rituximab.

## 1 Introduction

Immune-mediated necrotizing myopathies (IMNMs) are a group of rare idiopathic inflammatory myopathies (IIMs) characterized by severe muscle weakness, elevated muscle enzymes, and myonecrosis on muscle biopsy (1, 2); as with the majority of IIMs, there is a female predominance, with a female-to-male ratio of 3.2:1 (1, 2). Given the rarity of pediatric IMNMs and the small number of cases reported to date, the apparent discrepancy between the reported female predominance and our two patients is most likely attributable to the limited sample size rather than a different sex distribution in pediatric disease. The pathogenesis of IMNM involves autoantibodies targeting specific muscle proteins, including signal recognition particle (SRP) and 3-hydroxy-3-methylglutaryl-CoA reductase (HMGCR) (3), which correspond to 61.5% and 38.4% of pediatric cases, respectively (4). Nevertheless, around 13%–40% of cases do not present detectable antibodies, leading to a category of seronegative IMNM (4, 5). This wide range in prevalence is likely due to heterogeneity in cohort composition and diagnostic criteria, differences in antibody assay sensitivity and availability, and the inclusion of historical cohorts evaluated before the widespread implementation of current anti-SRP and anti-HMGCR testing. IMNM showed a clinical phenotype characterized by proximal muscle weakness, often with a limb-girdle distribution, variably associated with myalgias and axial involvement. In some cases, cardiac and bulbar dysfunction can occur (3, 6). The typical myopathological pattern reveals features of myocytes at different stages of necrosis, with deposition of the membrane attack complex (MAC) on the surface of non-necrotic myocytes, usually without a significant inflammatory infiltrate (7). These conditions are typically seen in adults but can appear in children with early onset and severe progression, raising suspicion for other neuromuscular disorders, such as early-onset muscular dystrophies (1, 7–9). A comprehensive clinical, serological, and paraclinical characterization is essential for identifying pathogenic autoantibodies, while muscle biopsy and deep genetic analysis remain two key tools for investigating more complex or seronegative cases.

This study presents an analysis of two male pediatric patients with IMNM, who presented with symptom onset at 2 and 16 years of age and received definitive diagnoses at 5 and 17 years, respectively. These patients were followed and treated at Raymond Poincaré and Henri Mondor hospitals of the APHP in France. The clinical and paraclinical data were obtained from medical reports. P1 and P2 both underwent an open deltoid muscle biopsy after parental informed consent. All samples were processed according to standard histochemical and histoenzymatic procedures described in Paternostro-Sluga et al. (10). The myopathological analysis was performed by some of the authors (E.M. and F.A.). Both patients underwent whole-body muscle MRI, and the images were analyzed by a radiologist specializing in neuromuscular disorders. T1-weighted and water-T2 sequences were used to assess fibro-fatty replacement and muscle inflammation or myoedema, respectively. After parental informed consent, both patients underwent whole-exome sequencing (WES) and *in silico* panel analysis to investigate variants in 298 genes implicated in muscle pathology in genomic DNA from peripheral blood, according to the French and Italian genetic diagnosis laboratories. Variants were interpreted and classified according to the 2015 American College of Medical Genetics (ACMG) Standards and Guidelines (11).

## 2 Case reports

**Figure 1:**
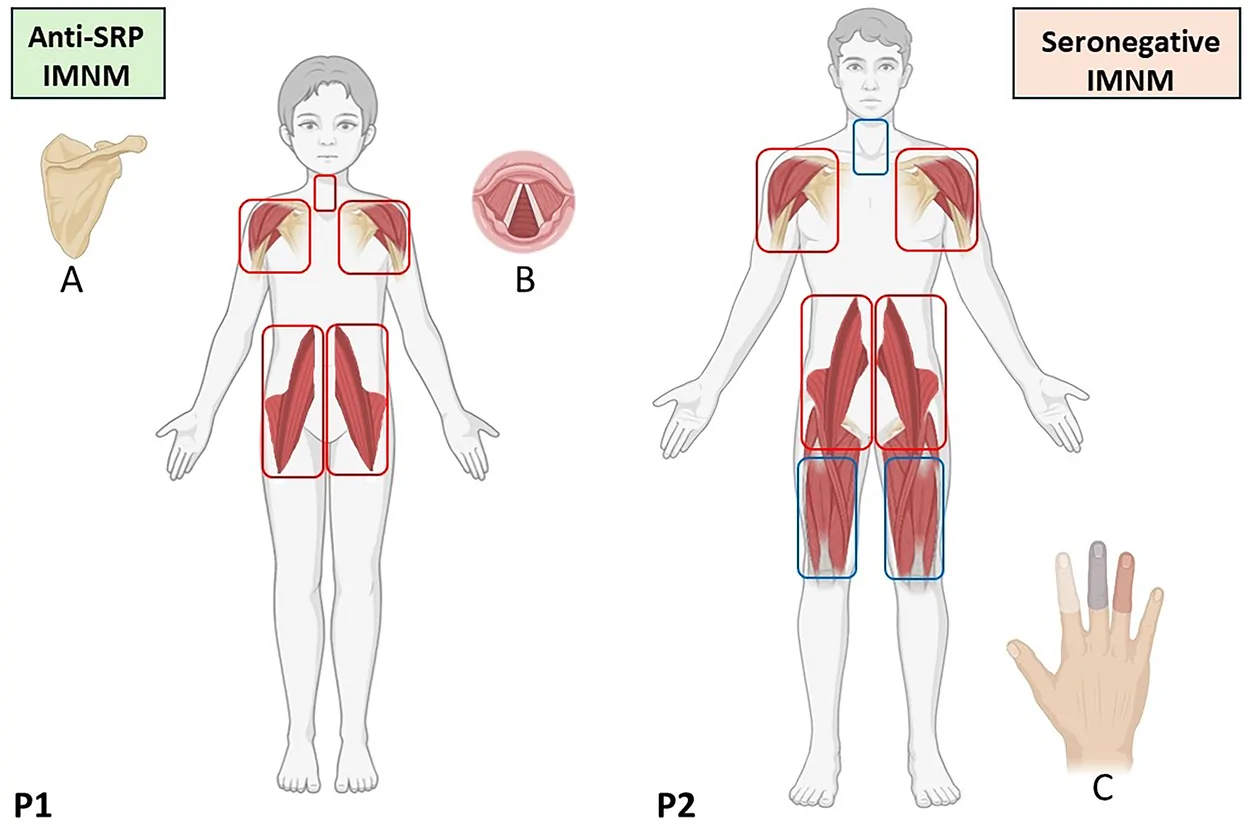
Clinical features at disease onset. The red outlines indicate areas of major muscle involvement (2–3/5 MRC), while the blue outlines indicate intermediate muscle involvement (4/5 MRC). P1 showed severe proximal and axial muscle weakness associated with *scapula alata* **(A)** and a high-pitched voice **(B)**. P2 revealed severe proximal (red outlines) and moderate axial and thigh muscle weakness (blue outlines) associated with Raynaud's phenomenon **(C)**. This figure was created using biorender.com (license provided by EM).

The main clinical features of P1 and P2 are depicted in Figure 1.

### 2.1 Case 1

**Figure 2:**
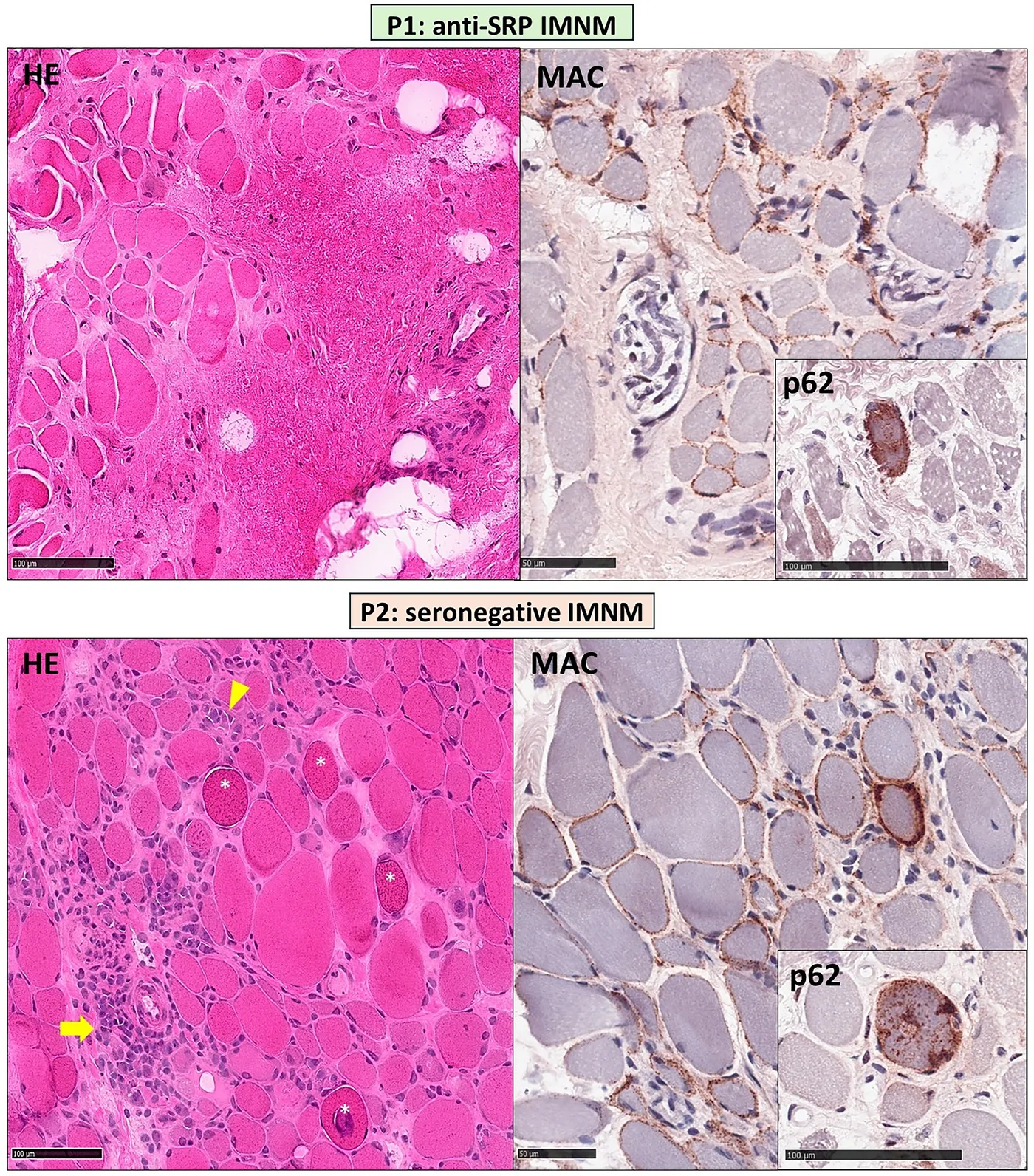
Myopathological pattern. HE, hematoxylin and eosin; MAC, membrane attack complex; p62. P1 (anti-SRP IMNM): HE: marked fibrosis, fiber-size variability, and rounded fiber atrophy. MAC: a large number of non-necrotic muscle fibers showed granular MAC deposition on the sarcolemma. p62: intracytoplasmic p62-immunoreactive aggregates in several muscle fibers. P2 (seronegative IMNM): HE: fiber-size variability, endomysial fibrosis, myophagocytosis (yellow arrowhead), hypercontracted fibers (white star), and inflammatory infiltrates (yellow arrow). MAC: prominent MAC deposition on the sarcolemma of non-necrotic muscle fibers. p62: intracytoplasmic p62-immunoreactive aggregates in several muscle fibers.

P1 is a 5-year-old Algerian boy born to non-consanguineous, healthy parents with no family history of neuromuscular disorders and normal motor development. At 2 years and 6 months of age, he was hospitalized for a COVID-19 infection presenting with fever and respiratory symptoms. Routine labs showed elevations in liver enzymes [Alanine aminotransferase (ALT) 123 IU/L and Aspartate aminotransferase (AST) 241 IU/L] and a creatine kinase (CK) level of 7,620 IU/L. Clinical examination revealed proximal muscle weakness that resolved completely after the infection; despite clinical restoration, the CK level remained elevated between 3,407 and 1,062 IU/L. Over time, the patient developed progressive muscle weakness, fatigability, and weight loss, leading to difficulty with previously acquired milestones, such as running and jumping, accompanied by frequent falls. By the age of 4, he required assistance with daily activities. A neuromuscular examination at 5 revealed global muscular atrophy and hypotonia, associated with a waddling gait and hyperlordosis. Facial and bulbar examination showed only an asymmetric smile, *micrognathia*, and a high-pitched voice, with no dysphagia or extraocular muscle weakness/diplopia. At the axial level, severe bilateral *scapula alata* and marked weakness of the neck flexors, rated 2/5 on the Medical Research Council (MRC), were noted in the absence of scoliosis or joint contractures (10). P1 showed severe and symmetric proximal muscle weakness, with deltoids, glutei, and psoas muscles rated 2/5, alongside a positive Gowers' sign. P1’s screening for antinuclear antibodies (ANAs) using indirect immunofluorescence (IIF) showed high titers, and further testing for myositis-associated autoantibodies and myositis-specific autoantibodies revealed strong reactivity to anti-SRP antibodies. Screening for anti-SRP and/or anti-ribosome antibodies by IIF on triple substrate confirmed the initial high-titer positivity. P1 did not exhibit palpitations, signs of heart failure, or respiratory symptoms. Echocardiographic findings were unremarkable. P1 underwent a deltoid muscle biopsy at the age of 6. The myopathological analysis revealed marked fiber-size variability and rounded atrophy, accompanied by endomysial and perimysial fibrosis. Immunohistochemistry showed multiple regenerating fibers, macrophagic infiltrates, and upregulation of HLA class I antigens on myocyte surfaces. Granular, sarcolemmal deposition of the MAC was observed in most non-necrotic myocytes, alongside p62 granular cytoplasmic aggregates in various myocytes. Overall, the muscle biopsy showed a dystrophic pattern associated with partial re-expression of MHC-I and diffuse complement deposits (Figure 2).

**Figure 3:**
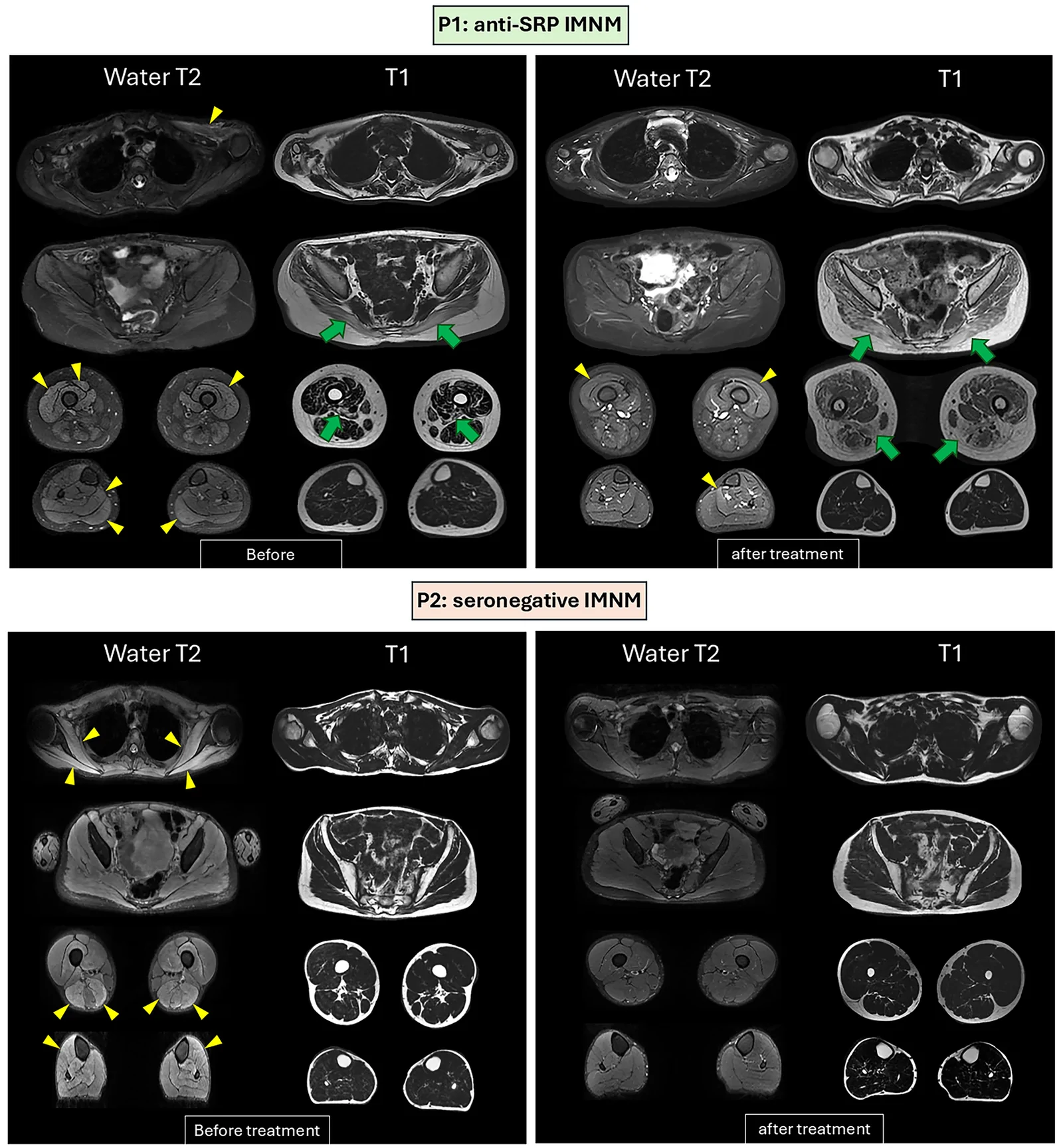
Muscle imaging features. Whole-body muscle MRI (axial T1-weighted and water-T2 sequences). P1 (anti-SRP IMNM): Before treatment, images show intense water-T2 muscle hypersignal (yellow arrowhead) and variable fibro-fatty replacement on T1-weighted sequences, especially in the bilateral *gluteus maximus* and *adductor magnus* muscles (green arrows). After treatment, there is persistence of water-T2 hypersignal in the lower limbs (yellow arrowhead) and worsening of fibro-fatty replacement on T1-weighted sequences (green arrows). P2 (seronegative IMNM): before treatment, images show diffuse water-T2 hypersignal, prominent in the periscapular muscles, posterior thigh compartment, and anterior leg compartment (yellow arrowhead), with no alterations on T1-weighted sequences. After treatment, there is global regression of the muscle hypersignal.

P1 received a whole-body muscle MRI at the age of 5. Water-T2 sequences revealed multifocal muscle hypersignal corresponding to inflammation and muscle edema. In the upper limb, the left *pectoralis* muscle showed an asymmetric hypersignal compared to the opposite side. At the thigh level, severe bilateral involvement of the *quadriceps* muscles was noted, with asymmetric involvement of the *rectus femoris.* At the leg level, diffuse muscle hypersignal was noted, which was more prominent in the posterior compartment, involving the *soleus* and *gastrocnemius medialis*. T1-weighted sequences revealed areas of fibro-fatty replacement in muscles showing water-T2 hypersignal, such as left *pectoralis* and bilateral *quadriceps*, and less prominently in the posterior compartment of the leg, alongside symmetrical involvement of the *gluteus maximus*, *adductor magnus*, and posterior compartment of the thigh. After 1 year and 5 months of treatment, P1 received a second whole-body muscle MRI, which showed persistent short tau inversion recovery (STIR) hypersignal, especially in the quadriceps and the posterior compartment of the legs. Meanwhile, T1-weighted sequences revealed an increase in symmetrical fibro-fatty replacement, notably in the glutei, *iliopsoas*, and thigh muscles (Figure 3).

In this study, we used WES and *in silico* panel analysis to investigate variants in 298 genes implicated in muscle pathology. The identification of variants in genes such as *SYNE1*, S*OX8, MYH3*, *PIEZO2*, and *TNFRSF13C* provides valuable insights into the genetic basis of P1 muscle pathology (Supplementary Table S1). Previous studies have indicated that *SOX8* plays a critical role in the transition from progenitor cells to differentiated muscle fibers (11). WES identified the heterozygous *SOX8* variant c.688A>C, p.(Thr230Pro). This missense variant is absent from the gnomAD population database and is classified as a variant of uncertain significance (VUS) according to the American College of Medical Genetics and Genomics/Association for Molecular Pathology (ACMG/AMP) criteria. *In silico* predictions were variable and did not provide sufficient evidence to support pathogenicity. *SOX8*-related congenital myopathy has mainly been associated with biallelic variants, and P1 did not exhibit the complete syndromic phenotype; therefore, this variant was considered an incidental finding.

### 2.2 Case 2

P2 is a 17-year-old Senegalese boy born to non-consanguineous parents, with a history of normal motor development. His family history is notable for a 14-year-old brother diagnosed with sclerodermatomyositis and anti-fibrillarin antibodies, presenting with a clinical onset of dermatological symptoms, including small, pigmented spots on the thoracic region, Raynaud's phenomenon, and episodes of myalgia unrelated to physical activity, without accompanying muscle weakness. P2’s brother is currently treated with prednisone (5 mg/day) and mycophenolate mofetil (750 mg twice a day), showing a favorable clinical evolution. Our patient presented at 16 years of age with subacute, progressive muscle weakness leading to difficulty walking, running, and performing daily activities. He sought medical attention due to the progressive worsening of symptoms and the onset of Raynaud's phenomenon, with no other additional features within the scleroderma spectrum. Biological testing showed rhabdomyolysis with a CK level of 17,467 IU/L, while metabolic and toxicological profiles were normal. At the initial neuromuscular examination, P2 exhibited a waddling gait and moderate proximal myalgias, while facial and bulbar functions were normal. Mild axial weakness was noted, with neck flexors graded 4/5 on the MRC scale, in the absence of scoliosis or signs of a rigid spine. Severe, bilateral proximal weakness of the upper limb was noted, with the deltoids graded 2/5, whereas the lower limbs showed moderate, symmetrical weakness in the psoas (3/5), quadriceps (4/5), and hip adductors and abductors (3/5). The clinical presentation was that of a subacute myopathy with a limb-girdle and axial distribution of muscle weakness. P2’s serological analysis showed high-titer positivity for ANAs with a speckled pattern and anti-Sjögren's syndrome-related antigen A (anti-SSA) 60 kDa specificity, while myositis-associated autoantibodies and myositis-specific autoantibodies (including anti-HMGCR and anti-SRP) were negative across two different assays. No anti-fibrillarin, anti-centromere, or anti-RNA polymerase III antibodies were detected. P2 underwent an open deltoid muscle biopsy at the age of 16, which revealed a severe dystrophic pattern characterized by marked fiber-size variability, endomysial fibrosis, inflammatory infiltrates, fibers with nuclear internalization, and myocytes at various stages of necrosis, such as hypercontracted fibers and histological signs of myophagocytosis. Immunohistochemistry showed sarcolemmal absence of dysferlin, with only rare positive fibers, along with diffuse upregulation of MHC-I on the myocyte surface. MAC deposition was observed on the surface of multiple non-necrotic fibers, and p62 granular cytoplasmic aggregates were present in various myocytes (Figure 2). Western blot analysis showed preserved dysferlin protein expression, although alterations in calpain-3 isoforms were identified: only trace amounts of full-length calpain-3 (94 kDa) and the minor autolytic fragment (30-kDa) were detected, whereas the major autolytic fragment (60 kDa) was preserved.

A whole-body muscle MRI at the age of 16 showed severe, diffuse muscle hypersignal on water-T2 sequences, predominantly affecting the periscapular muscles, posterior forearms, and bilateral long head of the *biceps femoris*, *semimembranosus*, and *tibialis anterior* muscles. No areas of fibro-fatty replacement were detected on T1-weighted sequences. After 7 months of treatment, a second whole-body muscle MRI showed complete normalization of the muscle hypersignal (Figure 3).

WES analysis identified variants in several genes associated with muscle pathology, namely, *SYNE1*, *CACNA1S*, and *SCN4A* (Supplementary Table S1). The variant identified in *CACNA1S* gene is widely recognized as pathogenic. *CACNA1S* encodes the alpha-1 subunit of the skeletal muscle voltage-gated calcium channel, which is essential for the initiation of muscle contraction. Pathogenic variants in this gene are known to disrupt calcium influx into muscle cells, resulting in a myriad of clinical manifestations, including hypokalemic periodic paralysis, malignant hyperthermia, and congenital myopathy. Traditionally, *CACNA1S* mutations have been associated with episodic weakness and paralysis; however, our findings suggest a more chronic phenotype that may not fit neatly into established diagnostic categories (12).

## 3 Comparative summary and treatment timeline

After the diagnosis of anti-SRP IMNM in P1, prompt therapy including corticosteroids and IVIG was started with partial clinical improvement and reduction of CK level. After the start of Rituximab, the CK level remained stable but never normalized, and was associated with only partial clinical amelioration. Recently, a combined treatment of azathioprine (AZA) and low-dose steroids was started (Table 1).

**Table 1:** Comparative summary**.**

(A)		
Feature	P1	P2
Onset (years)	2.5 y	16 y
Phenotype	Severe proximal/axial weakness, atrophy, scapular winging, Gowers +	Axial and limb girdle weakness, myalgia;, Raynaud phenomenon
Peak CK	7,620 IU/L	17,467 IU/L
Anti-HMGCR	-	-
Anti-SRP	+++	-
ANA	high titer	high titer (anti-SSA60)
Biopsy	Dystrophic/necrotizing pattern, fibrosis, regeneration	Severe dystrophic/necrotizing pattern, inflammation, myophagocytosis
IHC/WB	MHC-I ↑; MAC+++ non-necrotic fibers, p62++ aggregates	MHC-I ↑, MAC+++ non necrotic fibers, p62++ aggregates, dysferlin absent on IHC but preserved on WB; secondary CAPN3 reduction
MRI baseline	Water-T2 oedema + T1 fibro-fatty replacement	Diffuse Water-T2 oedema, no T1 fibro-fatty replacement
MRI follow-up	Persistent oedema, increased fibro-fatty replacement	Complete oedema resolution after 7 months
Diagnosis	Anti-SRP-positive IMNM	Seronegative IMNM

MN, immune-mediated necrotizing myopathy; MHC, Major Histocompatibility Complex class I; IHC, immunohistochemistry; MAC, membrane attack complex; WB, Western blot.

**Table:** 

(B)				
Patient	Disease phase	Treatment	Dose/regimen	Clinical response
P1	Initial phase	Corticosteroids	NA	Partial improvement
		IVIG	2 g/kg	
	Maintenance phase	Rituximab	375 mg/m^2^ IV	Partial improvement
	Ongoing	Azathioprine + low-dose steroids	NA	Stabilization
P2	Initial phase	Corticosteroids	0.75 mg/kg/day	Complete clinical recovery
		IVIG	2 g/kg × 5 cycles	
	Maintenance phase	Steroid taper	8 mg/day	Relapse
	At relapse	Steroid re-escalation	0.75 mg/kg/day	Partial improvement
	Ongoing	Rituximab	375 mg/m^2^ IV every 6 months	Complete clinical recovery

NA, not available; IVIG, intravenous immunoglobulin.

The therapeutic approach for P2 included corticosteroid therapy at 0.75 mg/kg daily, resulting in a partial reduction in the CK level and clinical improvement in fatigability, without clinically meaningful improvement on the MRC scale. IVIG at a dose of 2 g/kg over 3 days for five consecutive cycles was added, leading to improvement in muscle strength (5/5 on MRC) and normalization of CK. The corticosteroid dosage was gradually tapered to 8 mg/day, which led to a relapse of weakness accompanied by a CK level of 6,657 IU/L, requiring an increase in corticosteroids to 0.75 mg/kg (43 mg). Due to steroid dependence, following acute-phase treatment with IVIG, rituximab was initiated at 700 mg for four cycles (Table 1). Currently, the patients have normalized CK levels, exhibit no muscle weakness, and show resolution of muscle inflammation on MRI. CK levels over time and their relationship to treatment response are shown in Supplementary Figure S1.

## 4 Discussion

IMNM is a rare immune-mediated disorder that may affect both adult and pediatric patients (3, 4). Pediatric IMNM, much like adult forms, clinically presents with insidious and progressive proximal and axial muscle weakness affecting both the upper and lower limbs (1–4). The clinical phenotype varies according to the serological status of the patient. Anti-SRP-mediated IMNM often features axial and bulbar weakness, along with more frequent extra-skeletal muscle damage, such as cardiac involvement or interstitial lung disease. Furthermore, anti-SRP IMNM has been associated with a more severe disease course in adult patients than anti-HMGCR positive and seronegative forms (4, 13, 14). However, evidence on prognostic differences among serological subtypes in pediatric IMNM remains limited, and whether these observations can be extrapolated to children is currently uncertain. In this work, we present two cases of pediatric IMNM—anti-SRP and seronegative—mimicking a genetic myopathy. Seronegative IMNM presents similarly to seropositive forms, characterized by proximal muscle weakness and rare cardiac impairment (15). The early onset of the disease and the distribution of muscle weakness, combined with high CK levels, raise clinical suspicion of an early-onset muscular dystrophy.

P1 presented an early onset of severe bilateral and symmetric proximal and axial muscle weakness, associated with *scapula alata*, major muscle atrophy, and joint contractures. The clinical phenotype and persistently elevated CK level were compatible with an early-onset limb-girdle muscular dystrophy (LGMD). The diagnosis of IMNM for P1 was made at age 6 after testing for myositis-associated autoantibodies and myositis-specific autoantibodies, which showed a strong reactivity to anti-SRP antibodies. P2 presented at the age of 16 with progressive limb-girdle and axial muscle weakness associated with rhabdomyolysis, in the absence of major muscle atrophy or joint contractures. Screening for myositis-associated autoantibodies and myositis-specific autoantibodies was negative. This clinical phenotype, combined with elevated CK levels and the absence of pathogenic antibodies, suggested a diagnosis of LGMD. In the context of severe, progressive muscle weakness, a comprehensive evaluation, including muscle imaging, myopathological analysis, and serological testing, is essential to avoid misdiagnosis between inherited and inflammatory idiopathic myopathies. This multi-parametric approach is especially critical given that laboratory parameters, such as creatine kinase levels, can occasionally present atypically or remain entirely normal despite severe, biopsy-proven muscle inflammation (16).

P1 received a deltoid muscle biopsy to characterize and quantify the muscle damage and avoid a misdiagnosis of an underlying genetic myopathy. Myopathological analysis revealed a severe, chronic disorder characterized by MAC deposits on the sarcolemma of non-necrotic fibers, consistent with the diagnosis of anti-SRP-mediated IMNM with normal expression of sarcolemmal proteins. Myopathological analysis of P2 revealed a dystrophic pattern with marked inflammatory infiltrates, MAC deposition on the surface of non-necrotic myocytes, and a diffuse absence of dysferlin (17) on immunohistochemistry, with only rare positive fibers. Dysferlinopathies are a group of inherited myopathies caused by pathogenic variants in *DYSF* gene, leading to a defective protein (18). In healthy skeletal muscle, dysferlin plays a major role in membrane repair, T-tubule function, and calcium homeostasis. One of the most frequent phenotypes of dysferlinopathies is LGMDR2, which presents with progressive proximal muscle weakness in the upper and lower limb and elevated CK levels (19, 20), consistent with clinical features of P2. Myopathological features of *DYSF* include the presence of inflammatory infiltrates, a dystrophic pattern, MAC deposition, and the absence of dysferlin (18). However, the Western blot analysis of the muscle sample from P2 showed normal expression of *DYSF* alongside a reduction in *CAPN3*; secondary reductions in *CAPN3* have already been described in association with *DYSF* pathogenic variants; because calpain-3 is a relatively labile and unstable muscle protease, it is particularly prone to degradation in the setting of marked inflammation and muscle-fiber necrosis (21). This important diagnostic pitfall may be related to the role of dysferlin as a calcium-dependent membrane-repair protein. The apparent loss of sarcolemmal dysferlin on immunohistochemistry may occur as a secondary phenomenon in the setting of extensive muscle-fiber necrosis and membrane injury associated with marked MAC deposition, rather than reflecting a primary dysferlinopathy. Another relevant myopathological feature that may help distinguish IMNM with a secondary dysferlin defect from LGMDR2 is the higher proportion of p62-positive fibers and the lower frequency of splitting fibers observed in necrotizing myopathy compared with dysferlinopathy (22).

Whole-body muscle MRI of P1 showed an imaging pattern compatible with IMNM, characterized by extensive water-T2 hypersignal accompanied by fibro-fatty replacement on T1-weighted sequences. Recently, Wang et al. (23) described a cohort of 55 pediatric patients with IMNM, including 11 with anti-SRP antibodies; in accordance with their data, our anti-SRP patient displayed prominent fibro-fatty replacement in the bilateral *gluteus maximus* and *adductor magnus* muscles (green arrows, Figure 3) and marked muscle edema in the *vastus lateralis.* Follow-up muscle MRI showed the evolution of fibro-fatty replacement despite clinical improvement and normalization of CK level under treatment. In the case of P2, whole-body muscle MRI was an essential tool in the differential diagnosis between IMNM and LGMD. As shown in Figure 3, P2 presented solely with diffuse muscle hypersignal on water-T2 sequences, indicative of muscle inflammation, without the fibro-fatty replacement on T1-weighted sequences normally seen in LGMDR2, which characteristically features early involvement of the posterior compartment of the thighs and legs, paraspinal muscles, and subscapularis (24). In the field of neuromuscular disorders, muscle MRI is a useful tool for differentiating between inflammatory and inherited muscle disorders; however, limited accessibility to muscle MRI restricts its use during the acute phase.

The management of pediatric IMNM requires prompt initiation of immunosuppressive therapies to prevent irreversible muscle damage (25). Both patients were started on steroids at diagnosis, leading to initial improvements in muscle strength and CK levels. P1 showed partial improvement with steroids and IVIG, but rituximab was subsequently introduced, which prevented further relapses; however, it did not fully restore motor function, a limitation previously described in patients with anti-SRP antibodies (5, 23, 25). P2 demonstrated a favorable response, achieving significant improvement following the addition of IVIG and Rituximab. Although the clinical course observed in our seronegative patient was favorable, this single observation should not be interpreted as evidence of a better prognosis or treatment responsiveness compared with anti-SRP-positive disease. This report has limitations, primarily related to the small number of patients included, which reflects the rarity of the disorder. Larger pediatric cohorts are required to clarify whether serological status influences the disease course and therapeutic response. Rituximab appears to be an effective option in refractory cases; however, optimal treatment protocols for pediatric patients with IMNM remain to be established (4, 25, 26).

## 5 Conclusion

This report contributes valuable insights into the presentation, diagnosis, and management of pediatric IMNM. The cases emphasize the importance of considering IMNM in the differential diagnosis of pediatric patients with progressive muscle weakness, elevated CK levels, and clinical or paraclinical suspicion of muscular dystrophy, thereby avoiding misdiagnosis and the undertreatment of an inflammatory myopathy. Wang et al. showed that within their cohort of 55 IMNM patients, 32.7% were initially misdiagnosed with limb-girdle muscular dystrophy (23). While autoantibody testing is essential for diagnosis, clinicians must remain aware that seronegative cases can occur and that muscle biopsy remains a critical diagnostic tool (13, 23, 27). In seronegative or more complex cases of IMNM presented with clinical and paraclinical features suggestive of muscular dystrophy, comprehensive genetic analysis, such as WES, can complete the investigation, and if negative, it further reinforces the necessity for prolonged immunosuppressive therapy, even in young patients. Early recognition and prompt treatment are essential to prevent long-term muscle damage and improve functional outcomes (3, 4, 8, 27). While the combination of corticosteroids, IVIG, and rituximab offers promise, further research is needed to refine treatment protocols and identify new therapeutic targets (5, 8, 27).

## Data Availability

The datasets presented in this article are not readily available because of ethical and privacy restrictions. Requests to access the datasets should be directed to the corresponding author.

## Supplementary material

SUPPLEMENTARY FIGURE S1 Therapeutic approach. Ck level evolution according to different treatment overtime. Each treatment is depicted with arrows of different colour. The thin yellow arrow indicates the low-dose steroid treatment. Dotted arrows represent the ongoing treatment. IVIG: Intravenous Immunoglobulin; RTX: Rituximab; AZA: Azathioprine.
